# Prototypical innate immune mechanism hijacked by leukemia-initiating mutant stem cells for selective advantage and immune evasion in Ptpn11-associated juvenile myelomonocytic leukemia

**DOI:** 10.21203/rs.3.rs-4450642/v1

**Published:** 2024-08-02

**Authors:** Hong Zheng, Peng Zhao, Zhenya Tan, Wen-Mei Yu, Juwita Werner, Elliot Stieglitz, Chris Porter, Shanmuganathan Chandrakasan, Daniel Wechsler, Simon Mendez-Ferrer, Cheng-Kui Qu

**Affiliations:** Department of Pediatrics, Aflac Cancer & Blood Disorders Center, Winship Cancer Institute, Children's Healthcare of Atlanta, Emory University School of Medicine, Atlanta, USA; Department of Pathophysiology, Anhui Medical University, Hefei, China; Department of Pediatrics, Aflac Cancer & Blood Disorders Center, Winship Cancer Institute, Children's Healthcare of Atlanta, Emory University School of Medicine, Atlanta, USA; Department of Pathophysiology, Anhui Medical University, Hefei, China; Department of Pediatrics, Aflac Cancer & Blood Disorders Center, Winship Cancer Institute, Children's Healthcare of Atlanta, Emory University School of Medicine, Atlanta, USA; Department of Pediatrics, Division of Pediatric Hematology-Oncology, University of California San Francisco, San Francisco, USA; Department of Pediatrics, Division of Pediatric Hematology-Oncology, University of California San Francisco, San Francisco, USA; Department of Pediatrics, Aflac Cancer & Blood Disorders Center, Winship Cancer Institute, Children's Healthcare of Atlanta, Emory University School of Medicine, Atlanta, USA; Department of Pediatrics, Aflac Cancer & Blood Disorders Center, Winship Cancer Institute, Children's Healthcare of Atlanta, Emory University School of Medicine, Atlanta, USA; Department of Pediatrics, Aflac Cancer & Blood Disorders Center, Winship Cancer Institute, Children’s Healthcare of Atlanta, Emory University School of Medicine, Atlanta, USA; Department of Hematology, Wellcome Trust-Medical Research Council Cambridge Stem Cell Institute, University of Cambridge, Cambridge, UK; Department of Pediatrics, Aflac Cancer & Blood Disorders Center, Winship Cancer Institute, Children's Healthcare of Atlanta, Emory University School of Medicine, Atlanta, USA

**Keywords:** JMML, PTPN11, Leukemia-initiating cell, Hematopoietic stem cell, Innate immunity, Inflammation, S100a9, S100a8

## Abstract

Juvenile myelomonocytic leukemia (JMML), a clonal hematologic malignancy, originates from mutated hematopoietic stem cells (HSCs). The mechanism sustaining the persistence of mutant stem cells, leading to leukemia development, remains elusive. In this study, we conducted comprehensive examination of gene expression profiles, transcriptional factor regulons, and cell compositions/interactions throughout various stages of tumor cell development in Ptpn11 mutation-associated JMML. Our analyses revealed that leukemia-initiating *Ptpn11*^*E76K/+*^ mutant stem cells exhibited *de novo* activation of the myeloid transcriptional program and aberrant developmental trajectories. These mutant stem cells displayed significantly elevated expression of innate immunity-associated anti-microbial peptides and pro-inflammatory proteins, particularly *S100a9* and *S100a8*. Biological experiments confirmed that S100a9/S100a8 conferred a selective advantage to the leukemia-initiating cells through autocrine effects and facilitated immune evasion by recruiting and promoting immune suppressive myeloid-derived suppressor cells (MDSCs) in the microenvironment. Importantly, pharmacological inhibition of S100a9/S100a8 signaling effectively impeded leukemia development from *Ptpn11*^*E76K/+*^ mutant stem cells. These findings collectively suggest that JMML tumor-initiating cells exploit evolutionarily conserved innate immune and inflammatory mechanisms to establish clonal dominance.

## INTRODUCTION

Juvenile myelomonocytic leukemia (JMML), a pediatric myeloproliferative neoplasm, manifests as a clonal hematopoietic disorder characterized by the excessive production of myeloid cells. This disease originates from driver mutations acquired in hematopoietic stem cells (HSCs) and is propagated and sustained by these mutated stem cells, known as leukemia-initiating cells^1–4^. JMML has limited therapeutic options. Relapse remains the primary cause of treatment failure, most likely due to the persistence of therapy-resistant, self-renewing leukemia-initiating cells^1–4^. Addressing this issue is crucial for improving treatment outcomes in JMML patients.

Genetically, JMML is associated with mutations in genes encoding signaling proteins involved in the RAS/ERK pathway, including *PTPN11*, *RAS*, *NF1*, *CBL*, and others.^1–4^. These mutations play a causal role in driving JMML development^5–8^. JMML arises from an HSC harboring a genetic mutation, yet the mechanisms by which the initially mutated stem cell (leukemia-initiating cell) acquires a competitive advantage and evades immune surveillance remain unexplored. Additionally, the specific reasons behind the propensity of disease-associated mutations to induce myeloid malignancy are not fully understood, and the molecular mechanisms governing the aberrant repopulation of these leukemia-initiating stem cells remain elusive. Understanding these mechanisms could illuminate strategies for therapeutically targeting and eliminating JMML initiating stem cells in established disease.

Of the genetic lesions identified in JMML, the protein tyrosine phosphatase *PTPN11* (SHP-2), a positive regulator of RAS signaling^9,10^, is the most frequently mutated (heterozygous)^11,12^. Mutations in *PTPN11* lead to a significant increase in the catalytic activity of SHP-2^12,13^. Patients carrying *PTPN11* activating mutations have the worst prognosis among all subtypes of JMML^14–17^. To elucidate the mechanisms underlying the pathogenesis of *PTPN11*-mutated JMML, our laboratory created a conditional *Ptpn11* allele in mice with the *Ptpn11*^*E76K*^ mutation, the most common *PTPN11* mutation found in JMML^11,12^, and developed an inducible disease model^6,18^. Induction of the *Ptpn11*^*E76K*^ mutation in the hematopoietic system resulted in a JMML-like myeloproliferative neoplasm with complete penetrance, affirming the causative role of this mutation in JMML^6^. In the present study, we take advantage of this unique disease model to investigate the cellular and molecular mechanisms involved in the pathological process of JMML following induction of the disease mutation. Our findings from single-cell transcriptomic profiling and experimental validations reveal an aberrant activation of innate immune responses in the mutated stem cells. These leukemia-initiating cells exploit innate immune and inflammatory mechanisms to gain a competitive advantage and evade anti-tumor immunity, ultimately leading to clonal dominance.

## RESULTS

### Aberrant activation of innate immune and inflammatory responses in leukemia-initiating Ptpn11^E76K/+^ stem cells.

To explore the intricate mechanisms of JMML pathogenesis, we conducted a comprehensive single-cell RNA sequencing (scRNA-seq) analysis on bone marrow (BM) cells isolated from mice with induced JMML (*Ptpn11*^*E76K*/+^/*Mx1-Cre*)^6^ and wild-type (WT, *Ptpn11*^*+/+*^*/Mx1-Cre*) control littermates. Utilizing gene expression pattern-based cell clustering, we identified 11 distinct cell clusters within the BM population on a t-distributed stochastic neighbor embedding (t-SNE) plot (Extended Data Fig. 1A). Clear distinctions among these clusters were evident in the heatmap representation of the expression patterns of the top 10 differentially expressed genes (DEGs) in each cluster (Extended Data Fig. 1B). Leveraging reference datasets^19,20^ permitted the identification of various hematopoietic cell types in different developmental stages, including HSCs, granulocyte-macrophage progenitors (GMPs), megakaryocyticerythroid progenitors (MEPs), monocytes, neutrophils, T cells, B cells, and others (Extended Data Fig. 1C). Cell type-specific signature genes were indeed well-represented in the identified cell clusters (Extended Data Fig. 1D). Notably, *Ptpn11*^*E76K*/+^ mutant HSCs (leukemia-initiating cells) and GMPs exhibited reduced abundance, while monocytes and neutrophils displayed an increase compared to their WT (*Ptpn11*^+/+^) counterparts (Extended Data Fig. 1C). The reduction of mutant stem cells/progenitors and the myeloid shift in hematopoietic cell development indicated hyperactivation of these leukemia-initiating cells and myeloid-committed progenitors. The decreased numbers of T cells and B cells in their hematopoietic systems suggested that the enhanced myeloid cell production resulted from skewed differentiation of *Ptpn11*-mutated stem cells. Gene set enrichment analysis (GSEA) demonstrated the upregulation of genes associated with immune processes and chemokine activities, particularly through the CC chemokine receptor (CCR), in *Ptpn11*^*E76K*/+^ mutant hematopoietic cells (Extended Data Fig. 1E).

**Figure 1:**
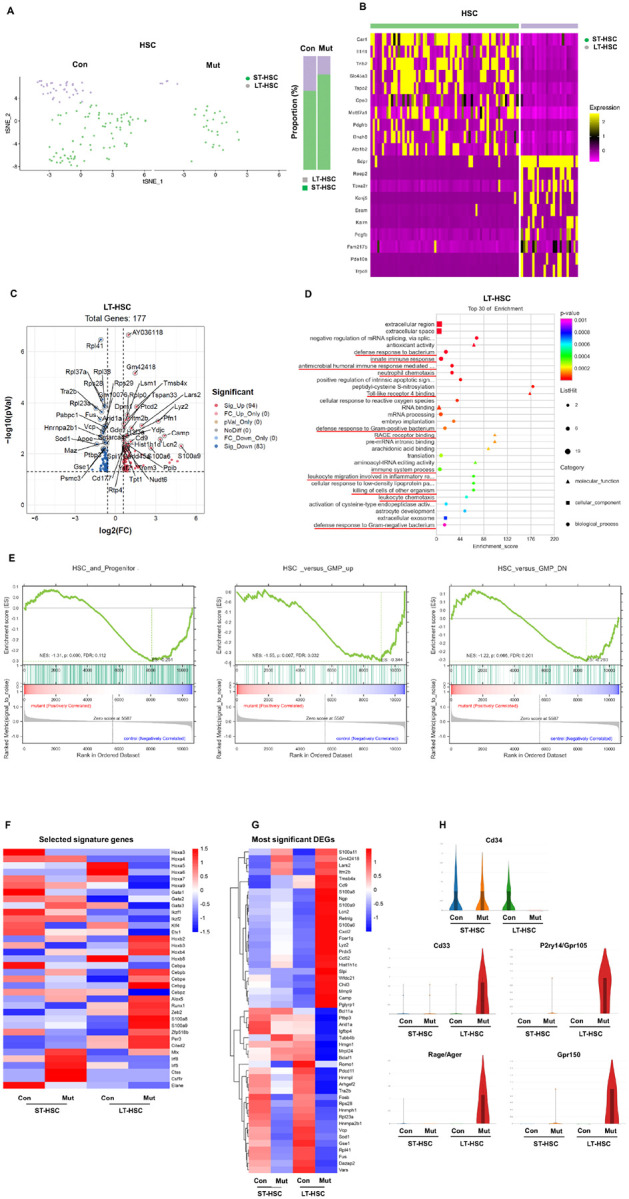
Aberrant activation of innate immune responses in *Ptpn11*^E76K/+^ mutant stem cells. **A.** HSCs were segregated into 2 distinct cell clusters equivalent to LT-HSCs and ST-HSCs based on their gene expression profiles. t-SNE plots of WT (*Ptpn11*^*+/+*^) and *Ptpn11*^*E76K*/+^ mutant HSCs are shown. **B.** Heatmap displaying the top 20 DEGs in cell clusters. Each column represents one cell, and relatively high and low expressions are represented in yellow and purple, respectively. **C.** Volcano plot depicting all DEGs in *Ptpn11*^*E76K*/+^ LT-HSCs compared to WT counterparts. DEGs with a fold change > 1.5 and a *p*-value < 0.05 were considered significant, with up-regulated genes labeled in red and down-regulated genes in blue. **D**. Significant DEGs in *Ptpn11*^*E76K*/+^ over WT LT-HSCs were subjected to Gene Ontology (GO) enrichment analysis, sorted based on *p* values, with *p*<0.05 considered significant. The size of the dots in the plot reflects the number of enriched genes in the respective terms, and the dot shape represents different GO classifications. **E.** Gene set enrichment analysis (GSEA) plots illustrating enrichments in the gene sets of HSC and progenitor, as well as HSC versus GMP up and down regulated in WT and *Ptpn11*^*E76K*/+^ HSCs. The y-axis represents the enrichment score (ES), and vertical blue lines on the x-axis indicate where genes annotated to the respective pathways appear in the ranked list of genes. The colored band represents ES values, with red indicating positive enrichment and blue indicating negative enrichment. **F.** Heatmap illustrating the expression of selected signature genes in WT and *Ptpn11*^*E76K*/+^ ST-HSCs and LT-HSCs. **G.** Heatmap displaying the expression of most significantly up or down regulated genes in WT and *Ptpn11*^*E76K*/+^ ST-HSCs and LT-HSCs. **H.** Violin plots illustrating the expression levels of the indicated cell surface markers in WT and *Ptpn11*^*E76K*/+^ ST-HSCs and LT-HSCs.

Gene expression profile-based cell clustering of the stem cell population revealed two distinct clusters equivalent to long-term HSCs (LT-HSCs) and short-term HSCs (ST-HSCs) according to the reference datasets^20^. The percentage of LT-HSCs decreased while the percentage of ST-HSCs increased in *Ptpn11*^*E76K*/+^ mice compared to those in WT littermates (Fig. 1A). In our analyses we also observed that among the top 20 DEGs in LT-HSCs compared to ST-HSCs, several genes were highly expressed only in LT-HSCs (Fig. 1B). In particular, *Sdpr* was predominantly expressed in LT-HSCs, indicating its potential as a distinctive marker for distinguishing LT-HSCs from ST-LT-HSCs. Notably, 177 genes in total were significantly differentially expressed in *Ptpn11*^*E76K*/+^ LT-HSCs versus WT LT-HSCs (Fig. 1C). The Gene Ontology (GO) enrichment analysis of these DEGs highlighted the predominant elevation of defense reactions to bacterial infection, innate immune response, Toll-like receptor 4 (TLR4) signaling, and Inflammation-associated pathways (Fig. 1D). Consistent with the hyperactivation of *Ptpn11*^*E76K*/+^ HSCs, GSEA demonstrated a decrease in the expression of stem cell/progenitor-associated genes and upregulated/downregulated genes in HSCs versus GMPs in *Ptpn11*^*E76K*/+^ HSCs (Fig. 1E), suggesting a loss of stemness and priming towards the myeloid lineage in Ptpn11-mutated HSCs. Similarly, 173 DEGs were identified in *Ptpn11*^*E76K*/+^ ST-HSCs compared to WT ST-HSCs (Extended Data Fig. 2A), with Kyoto Encyclopedia of Genes and Genomes (KEGG) enrichment analysis indicating dysregulation of anti-viral immune response pathways, ribosome biogenesis, and spliceosome function. (Extended Data Fig. 2B).

Further examination of stem cell self-renewal or differentiation-associated signature genes revealed widespread deregulation in *Ptpn11*^*E76K*/+^ LT-HSCs and ST-HSCs, as compared to WT counterparts (Fig. 1F). Notable downregulated genes in *Ptpn11*^*E76K*/+^ LT-HSCs included Hoxa5, Hoxa6, Hoxa7, and Hoxb8, while upregulated genes comprised *Hoxb2*, *Hoxb3*, and *Hoxb4*. Interestingly, upregulated expression of myeloid differentiation-related genes *Cebpb*, *Cebpe*, *Cebpg*, and *Cited2* was noticed in these mutant stem cells. In *Ptpn11*^*E76K*/+^ ST-HSCs, downregulated genes included *Hoxa3*, *Gata1*, *Klf4*, *Cebpa*, and *Elane*, while upregulated genes encompassed *Mix*, *Irf5*, *Irf8*, *Ctss*, *Gata3*, and *Csf1r*. The most significant DEGs in *Ptpn11*^*E76K*/+^ LT-HSCs and ST-HSCs versus WT counterparts are illustrated in Fig. 1G. Surprisingly, myeloid cell-specific genes and genes associated with anti-pathogen and innate immune responses normally activated in myeloid cells, such as *S100a9*, *S100a8*, *S100a6*, *S100a11*, *Retnlg*, *Ngp*, *Camp, Lcn2, Lyz2, Wfdc21, Chil3*, and *Pglyrp1* were highly expressed in *Ptpn11*^*E76K*/+^ LT-HSCs. The expression levels of *S100a9* and *S100a8*, also known as myeloid-related proteins 9 and 8, were increased approximately 29- and 24-fold, respectively, standing out as the most strikingly upregulated among all significant DEGs in *Ptpn11*^*E76K*/+^ LT-HSCs. Additionally, *Cxcl2*, also known as *MIP2*-α, a chemokine typically secreted by monocytes/macrophages and a powerful chemoattractant for polymorphonuclear leukocytes involved in many immune responses, including wound healing, cancer metastasis, and angiogenesis, was overexpressed in these leukemia-initiating cells.

Moreover, several cell surface molecules were differentially expressed in *Ptpn11*^*E76K*/+^ LT-HSCs. Among the most significant DEGs, *Cd52* and *Cd9* were upregulated, while transcriptional expression of the early stem/progenitor cell marker *Cd34* was diminished (Fig. 1H). In addition, *Cd33*, *P2ry14*/*Gpr105*, and *Gpr150* showed a marked upregulation in *Ptpn11*^*E76K*/+^ LT-HSCs. These unique expression patterns of cell surface molecules in *Ptpn11* mutant LT-HSCs hold promise for their utilization as therapeutic targets or biomarkers for JMML stem cells. Furthermore, Rage/Ager, the receptor for the S100a9/S100a8 heterodimer (calprotectin)^21^ typically expressed on myeloid immune cells exhibited substantial upregulation in *Ptpn11*^*E76K*/+^ LT-HSCs, indicating potential autocrine feedback activities in these leukemia-initiating cells. Given that *S100a9* expression was significantly upregulated in *Ptpn11*^*E76K*/+^ LT-HSCs (Extended Data Fig. 3A), we sought to identify transcriptional factors potentially associated with this upregulation. To this end, we conducted Venn diagram data analysis involving the 177 DEGs in *Ptpn11*^*E76K*/+^ LT-HSCs and 58 transcriptional factors related to *S100a9*. This analysis revealed *Spi1* and *Smarca4* (Extended Data Fig. 3B). Of the 47 dysregulated transcriptional factors in *Ptpn11*^*E76K*/+^ LT-HSCs, *Spi1* showed a significant upregulation, whereas Smarca4 was downregulated (Extended Data Fig. 3C), suggesting that 6the elevated levels of *Spi1* may have contributed to the observed overexpression of *S100a9*.

### Profound impact on the myeloid lineage by the Ptpn11^E76K^ mutation.

**Figure 2:**
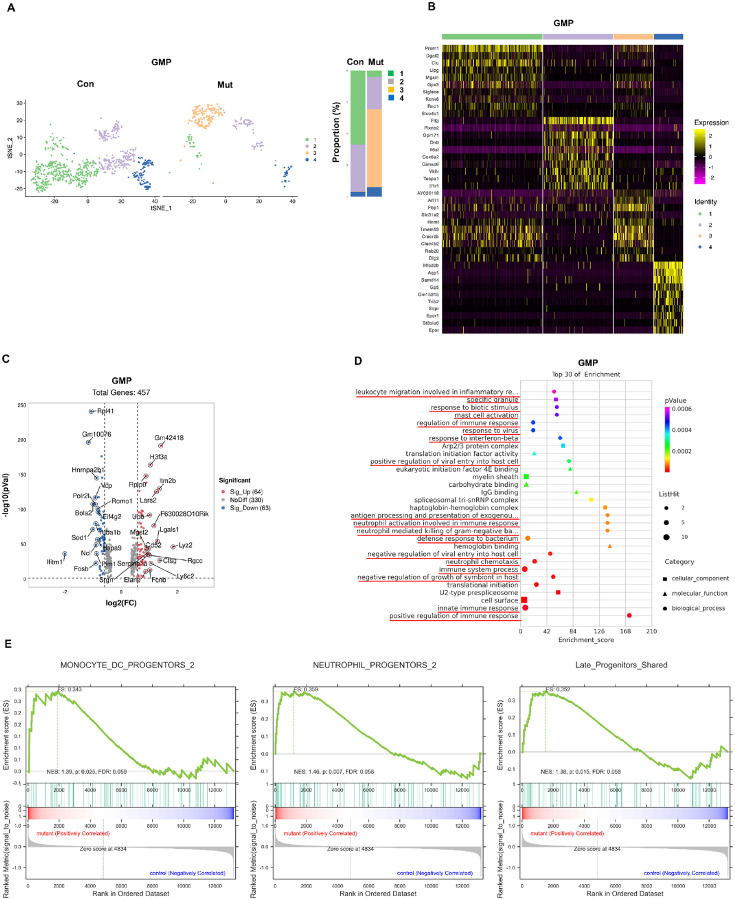
Enhanced innate immune signaling in *Ptpn11*^E76K/+^ granulocyte-macrophage progenitors (GMPs). **A.** GMPs were categorized into 4 different cell clusters based on their gene expression profiles. t-SNE plots of WT and *Ptpn11*^*E76K*/+^ mutant GMPs are displayed. **B.** Heatmap illustrating the expression patterns of the top 10 representative genes in each cell cluster. Each column represents one cell, and relatively high and low expressions are represented in yellow and purple, respectively. **C.** Volcano plot depicting DEGs in *Ptpn11*^*E76K*/+^GMPs. The DEGs with a fold change > 1.5 and a *p*-value < 0.05 are considered significant, with up-regulated genes labeled in red and down-regulated genes in blue. **D.** DEGs in *Ptpn11*^*E76K*/+^GMPs were subjected to GO enrichment analysis, sorted by *p* values, with *p*<0.05 considered significant. The size of the dots in the plot reflects the number of enriched genes in the respective terms, and the dot shape represents different GO classifications. E. GSEA plots depicting enrichments in gene sets of monocyte dendritic cell (DC) progenitors, neutrophil progenitors, and late progenitors-shared pathways in *Ptpn11*^*E76K*/+^ GMPs. The y-axis represents the ES, and vertical blue lines on the x-axis indicate where genes annotated to the respective pathways appear in the ranked list of genes. The colored band represents ES values, with red indicating positive enrichment and blue indicating negative enrichment.

The influence of the *Ptpn11*^*E76K*^ mutation extended beyond the stem cell population, significantly affecting myeloid-committed GMPs. Gene expression profiling identified 4 distinct cell clusters in GMPs, revealing heterogeneity among these progenitors (Fig. 2A). Interestingly, *Ptpn11*^*E76K*/+^ GMPs exhibited a notable shift in cell composition, with Cluster 3 emerging as a unique and overrepresented subpopulation, constituting approximately 60% of the total. The heatmap representation of the top 10 DEGs in each cell cluster highlighted clear differences among these clusters, with Cluster 1 enriched in *Prom1, Clu, Mgam, Gpx3*, and *Slco4c1*, and Cluster 3 marked by high expression of *Fbp1, Tmem53*, *Cracr2b*, and *Dlg2* (Fig. 2B). Overall, 127 genes were significantly differentially expressed in *Ptpn11*^*E76K*/+^ GMPs compared to their WT counterparts (Fig. 2C). GO enrichment analysis of the DEGs underscored enrichment in innate immune and inflammatory pathways in *Ptpn11* mutant GMPs (Fig. 2D). This included pathways related to the positive regulation of immune response, neutrophil activation, neutrophil-mediated killing of bacteria, defense response to bacteria, and innate immune response. GSEA revealed an enrichment of genes typically associated with later-stage progenitors, such as monocyte and dendritic cell progenitors, and neutrophil progenitors in *Ptpn11*^*E76K*/+^ GMPs relative to WT GMPs (Fig. 2E), indicative of enhanced differentiation activities in these mutant GMPs. Cluster 3, representing the major subpopulation within *Ptpn11*^*E76K*/+^ GMPs, displayed high and unique expression of *Arl11, Fbp1, Slc31a2, Hnmt, Tmem53, Cracr2b*, among others (Extended Data Fig. 4A). Differential gene expression analysis between Cluster 3 and Cluster 1, the major population in WT GMPs, revealed 114 genes with distinct expression patterns (Extended Data Fig. 4B). KEGG pathway analyses illustrated the upregulation of genes involved in autoimmune responses, bacterial infection responses, natural killer cell-mediated cytotoxicity, neutrophil extracellular trap formation, and ribosome, whereas downregulated pathways included phagosome, ribosome, RNA transport, spliceosome, RNA degradation, oxidative phosphorylation, and thermogenesis pathways in *Ptpn11*^*E76K*/+^ GMPs (Extended Data Fig. 4C).

**Figure 3:**
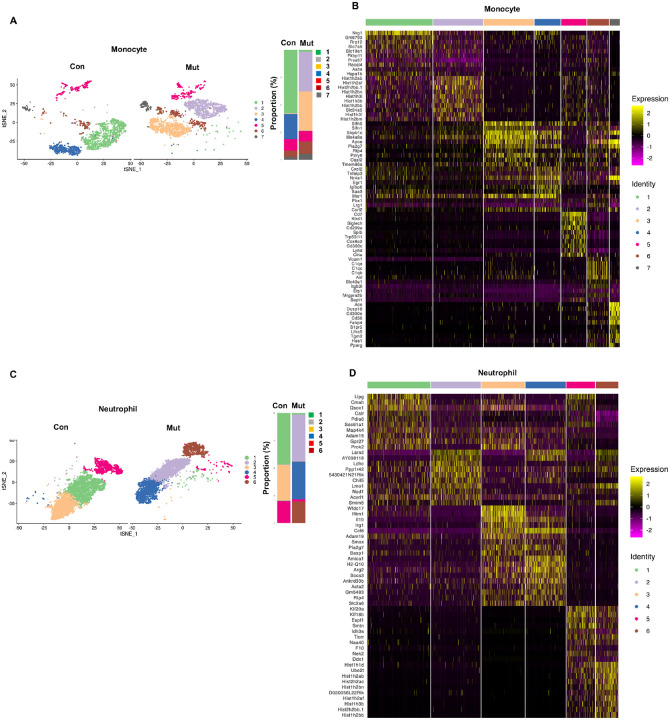
Impact of the *Ptpn11*^*E76K*^ mutation on monocytes and neutrophils. **A.** Monocytes were divided into 7 different cell clusters based on their gene expression patterns. t-SNE plots of WT and *Ptpn11*^*E76K*/+^ mutant monocytes are shown. **B.** Heatmap displaying the expression patterns of the top 10 representative genes in each cluster. Each column represents one cell, and relatively high and low expressions are represented in yellow and purple, respectively. **C.** Neutrophils were segregated into 6 distinct cell clusters according to their gene expression profiles. t-SNE plots of WT and *Ptpn11*^*E76K*/+^ mutant neutrophils are displayed. **D.** Heatmap illustrating the expression patterns of the top 10 representative genes in each cluster.

We then examined the impact of the *Ptpn11*^*E76K*^ mutation on monocytes and neutrophils. Gene expression profile-based cell clustering demonstrated heterogeneity in monocytes. Seven distinct cell clusters were identified in monocytes (Fig. 3A). The *Ptpn11*^*E76K*/+^ monocyte compartment demonstrated notable changes in cell compositions. The linker histone H1 family members (*Hist1h2ab, Hist1h2af, Hist1h2bm, Hist1h2bn, Hist1h3b*, and *Hist1h3f*), *Sirpb1c, Ms4a8a, Apoe, Slfn5, Pla2g7*, and *P2ry6* were highly expressed in Cluster 2 and Cluster 3, which were unique in the Ptpn11 mutant monocyte population (Fig. 3B). Similarly, neutrophils also exhibited heterogeneity, with altered cell compositions in the *Ptpn11*^*E76K*/+^ neutrophil compartment (Fig. 3C). Upregulated genes in *Ptpn11*^*E76K*/+^ clusters included mitochondrial protein synthesis-associated *Lars2*, innate immunity-associated *Chil5*, the chemokine *Ccl6*, Arginase, type 2 (*Arg2*), and glycolysis-associated *Ldhc*, while downregulated genes comprised *Lipg, Cmah, Qsox1, Calr, Pdia6, Sec61a1, Prok2, Wfdc17*, *Ifitm1*, and others (Fig. 3D).

**Figure 4:**
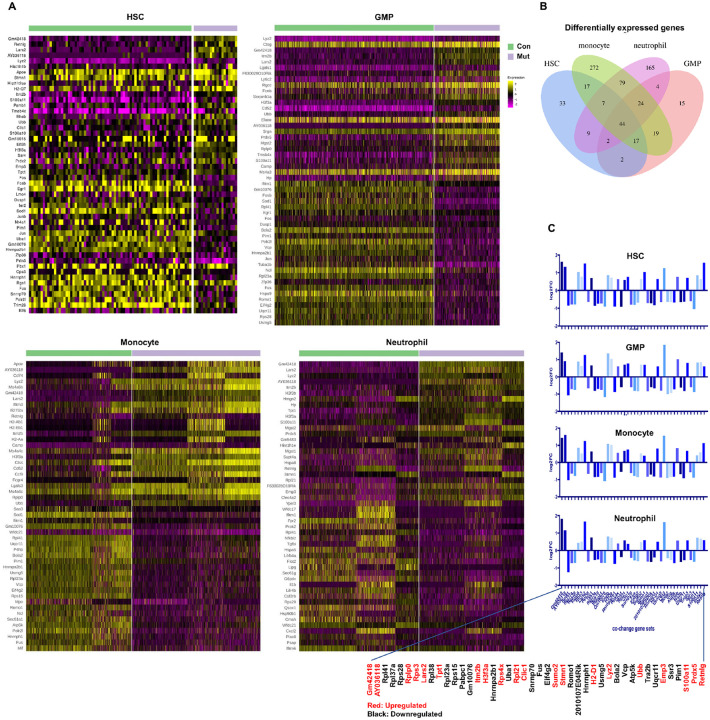
Effects of the *Ptpn11*^*E76K*^ mutation on ribosomes across developmental stages. **A.** Heatmaps depicting the expression of the genes that are up- or down regulated in *Ptpn11*^*E76K*/+^ HSCs, GMPs, monocytes, and neutrophils. Each column in the heatmaps represents one cell, and relatively high and low expression are represented in yellow and purple, respectively. **B.** Venn diagram data analysis was conducted on the DEGs in *Ptpn11*^*E76K*/+^ HSCs, GMPs, monocytes, and neutrophils. Different colors were superimposed to represent shared genes, and the numbers of these shared genes are indicated. **C.** Bar graphs illustrating the results of the differential expression analysis for the shared DEGs (44) in *Ptpn11*^*E76K*/+^ HSCs, GMPs, monocytes, and neutrophils. Up-regulated genes are marked in red, and down-regulated genes in black.

To explore whether the transcriptional landscape changes in *Ptpn11*^*E76K*/+^ cells across different developmental stages shared commonality, the top 50 significant DEGs in *Ptpn11*^*E76K*/+^ and WT stem cells, GMPs, monocytes, and neutrophils are shown in Fig. 4A. Venn diagram data analysis for DEGs in the different cell populations identified 44 co-events (Fig. 4B). Remarkably, these genes were consistently upregulated or downregulated in *Ptpn11*^*E76K*/+^ cells throughout all developmental stages, without any exceptions (Fig. 4C). This observation implies that they were cell-intrinsically dysregulated by the *Ptpn11*^*E76K*^ mutation. Many of these co-DEGs were associated with innate immune signaling and inflammatory pathways, including *S100a11*, *Retnlg*, and *Lyz2*. Interestingly, genes involved in ribosomal biogenesis, such as *Rplp0, Rps3*, and *Rpl21* were upregulated, while *Rpl41, Rpl37a, Rps28, Rpl38, Rpl23a*, and *Rps15* were repressed. Dysregulation of ribosomal biogenesis and function can collectively contribute to cellular abnormalities, genomic instability, and the development of malignancies^22,23^. These findings underscore that the impact on ribosomal function is a common pathological effect of the *Ptpn11* mutation across different cell types.

### Altered developmental trajectories and cell-cell communications in leukemia-initiating Ptpn11^E76K/+^ stem cells.

**Figure 5:**
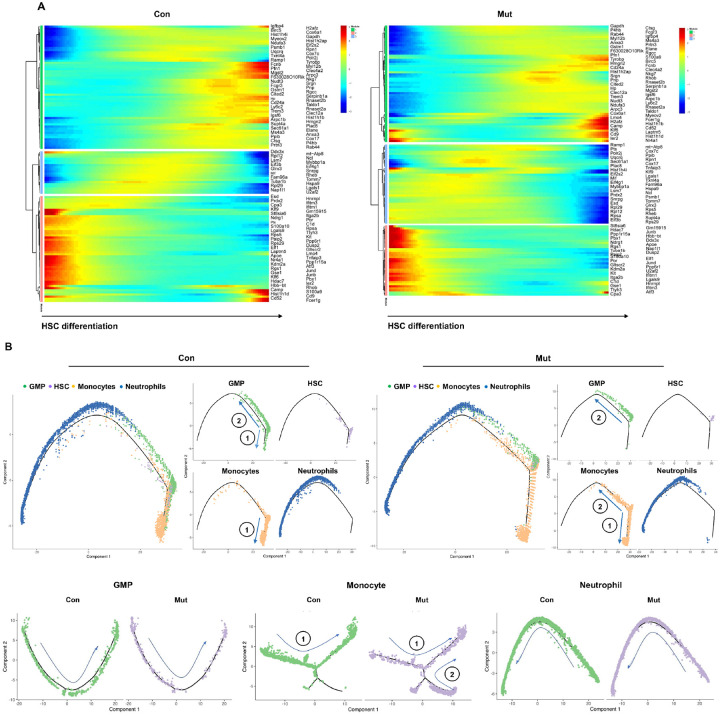
Altered developmental trajectories in *Ptpn11*^E76K/+^ mutant stem cells. **A.** Heatmaps of DEGs relevant to HSC differentiation in WT and *Ptpn11*^*E76K*/+^ mutant HSCs across the pseudo-time. The color gradient indicates the average expression, ranging from dark blue to red. **B.** Pseudo-time analysis using Monocle2 revealing the differentiation trajectory of WT and *Ptpn11*^*E76K*/+^ mutant HSCs to GMPs and further to monocytes/neutrophils (top). Pseudo-time analyses for WT and *Ptpn11*^*E76K*/+^ mutant GMPs, monocytes, and neutrophils were conducted separately (bottom). The directions of differentiation for each cell type are indicated by arrows.

Branched expression analysis modeling (BEAM), followed by hierarchical clustering analysis, identified three distinct gene expression modules during the differentiation process from stem cells to monocytes and neutrophils. Notable differences in the dynamic changes in the expression of genes enriched in all modules were observed in the differentiation process of *Ptpn11*^*E76K*/+^ stem cells (Fig. 5A). A markedly higher number of genes showed dynamic changes in expression within Module 2, whereas fewer genes demonstrated such changes in Module 3 in the context of the *Ptpn11* mutant cellular processes. Pseudotime mapping analysis, which infers the developmental trajectory or temporal progression of cells within a heterogeneous population based on gene expression profiles, revealed that leukemia-initiating *Ptpn11*^*E76K*/+^ mutant stem cells gave rise to GMPs mainly in one direction as opposed to two in WT counterparts (Fig. 5B, upper row), suggesting the impact on the mutation of GMPs. While the inferred pseudotime of neutrophil development from *Ptpn11*^*E76K*/+^ stem cells remained relatively unchanged, two diverging cell fates were observed during the differentiation of these leukemia-initiating cells towards monocytes, contrasting with the essentially singular fate observed in the WT control, and the inferred pseudotime of monocyte development from the leukemia-initiating cells was prolonged (Fig. 5B, upper row). In addition, intermediate monocytes in a transitioning state were increased in the *Ptpn11*^*E76K*/+^ group, suggesting a delay or arrest in their differentiation and maturation. Further analyses focusing on specific cell compartments showed a slight difference in the diffusion trajectories within GMPs between *Ptpn11*^*E76K*/+^ and WT counterparts (Fig. 5B, lower row). No notable differences in *Ptpn11*^*E76K*/+^ neutrophil diffusion maps were detected, indicating relatively normal differentiation and maturation within these two cell populations. In contrast, *Ptpn11*^*E76K*/+^ monocytes exhibited two distinct developmental paths compared to the single direction observed in WT monocytes (Fig. 5B, low row), implying the generation of various subpopulations in *Ptpn11*^*E76K*/+^ monocytes along distinct developmental routes.

Cell-cell communication analyses based on the expression of ligands and their cognate receptors revealed enhanced interactions between neutrophils and stem cells in *Ptpn11*^*E76K*/+^ mice compared to those in WT littermates (Extended Data Fig. 5A and 5B). Furthermore, interactions among *Ptpn11*^*E76K*/+^ stem cells were increased relative to those in WT stem cells (Extended Data Fig. 5A and 5B). A closer examination of neutrophil-stem cell communications indicated that interactions mediated by IL-1β, TGF-β, and Oncostatin M were enhanced in *Ptpn11*^*E76K*/+^ mice compared to those in WT mice (Extended Data Fig. 5C), providing additional evidence that leukemia-initiating *Ptpn11*-mutated stem cells were situated in an inflammatory microenvironment.

### Leukemia-initiating Ptpn11^E76K/+^ stem cells are primed by the myeloid transcriptional program.

**Figure 6:**
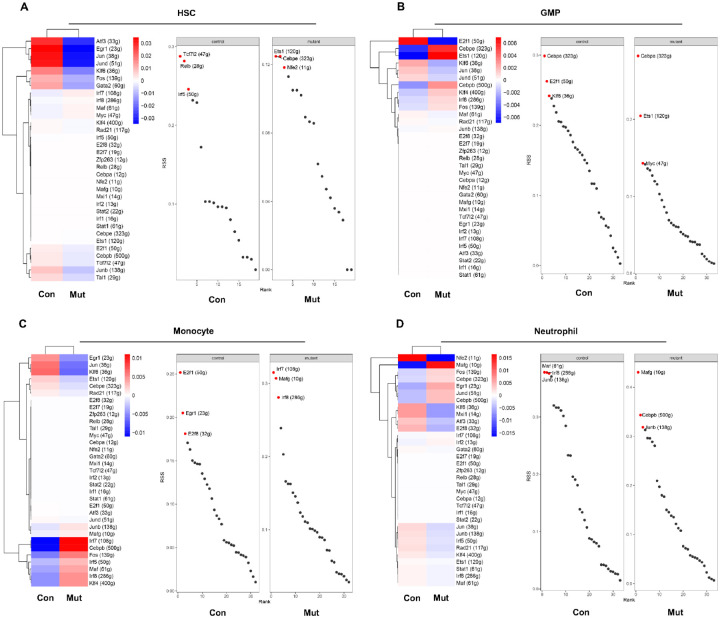
*De novo* activation of the myeloid transcriptional program in *Ptpn11*^E76K/+^ mutant stem cells. **A-D.** Heatmaps displaying the results of SCENIC analysis, revealing the regulatory activities of transcription factors in WT and *Ptpn11*^*E76K*/+^ HSCs (**A**), GMPs (**B**), monocytes (**C**), and neutrophils (**D**) are shown on the left. Functionally up-regulated transcription factors are indicated in red, with down-regulated transcription factors in blue. The top three cell specificity-determining regulons with highest regulon specificity score (RSS) in WT and *Ptpn11*^*E76K*/+^ HSCs (**A**), GMPs (**B**), monocytes (**C**), and neutrophils (**D**) are shown on the right.

Cell identity and functional specificity are collectively governed by transcription factors and the expression levels of their target genes. The overall transcriptional activities in *Ptpn11*^*E76K*/+^ stem cells were elevated compared to those in their WT counterparts (Extended Data Fig. 6), consistent with more active cellular processes in leukemia-initiating Ptpn11-mutated stem cells. To further elucidate the mechanisms through which the *Ptpn11*^*E76K*^ mutation influences cell behavior, we conducted single cell regulatory network inference and clustering (SCENIC) analysis (transcriptional factor regulon analysis). The activities of many transcription factors in *Ptpn11*^*E76K*/+^ stem cells, GMPs, monocytes, and neutrophils were altered compared to those in their WT counterparts, as indicated by regulon activity scores. In *Ptpn11*^*E76K*/+^ stem cells, the transcriptional activities of *Atf3, Egr1, Jun, Jund, Klf6, Fos*, and *Gata2* were significantly decreased, while those of *Irf7, Irf8, Maf*, and *Myc* were increased (Fig. 6A). Importantly, regulon specificity scores (RSS), reflecting the association between regulon activities and cellular specificity, revealed that among these differentially functioning transcription factors, the myeloid transcription factors *Ets1, Cebpe*, and *Nfe2* were highly associated with the identity specificity of *Ptpn11*^*E76K*/+^ stem cells, as opposed to *Tcf7l2, Relb*, and *Irf5* for WT HSCs. At the GMP level, the activities of myeloid-specific transcription factors *Cebpe, Cebpb*, and *Ets1* were markedly increased in *Ptpn11*^*E76K*/+^ GMPs, and their cellular specificity was determined by Cebpe, Ets1, and Myc compared to *Cebpe, E2f1*, and *Klf6* in WT GMPs (Fig. 6B). Activities of transcription factors *Irf7, Cebpb, Fos, Irf5, Irf8, Klf4*, and *Maf* were significantly higher in *Ptpn11*^*E76K*/+^ monocytes than those in WT cells, and the identity specificity of *Ptpn11* mutant monocytes was highly associated with transcription factors *Irf7, Mafg*, and *Irf8* according to RSS (Fig. 6C). Similarly, the distinction in transcriptional factor determinants influencing the specificity of *Ptpn11*^*E76K*/+^ neutrophils (*Mafg, Cebpb*, and *Junb*) compared to those governing WT neutrophils (*Maf, Irf8*, and *Junb*) was also apparent (Fig. 6D).

**Figure 7:**
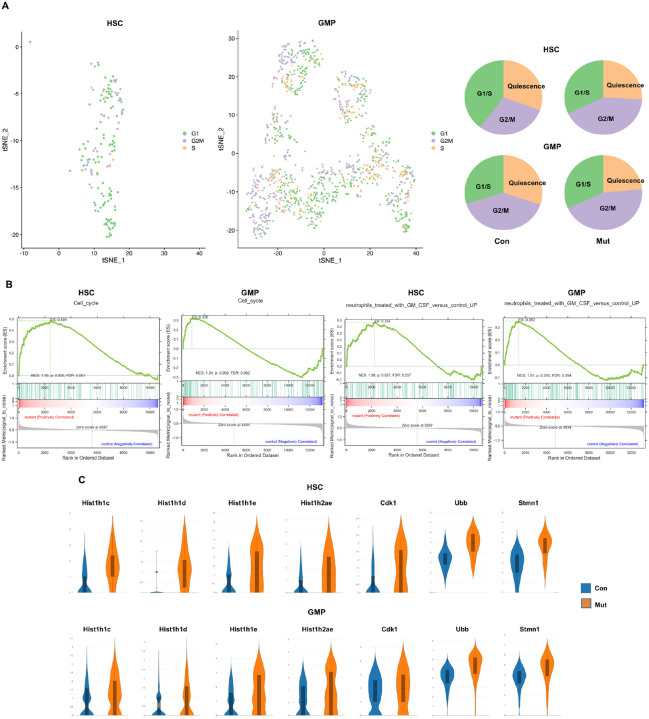
*Ptpn11*^E76K/+^ stem cells and GMPs exhibit enhanced cell cycling. **A.** t-SNE plots depicting the cell cycle distribution of HSCs and GMPs. The proportions of different cell cycle phases in WT and *Ptpn11*^*E76K*/+^ HSCs and GMPs are displayed on the right. **B.** GSEA plots illustrating enrichments in cell-cycling associated gene sets and GM-CSF responsive gene sets in *Ptpn11*^*E76K*/+^ HSCs and GMPs. The y-axis represents the ES, and vertical blue lines on the x-axis indicate where genes annotated to the respective pathways appear in the ranked list of genes. The colored band represents ES values, with red indicating positive enrichment and blue indicating negative enrichment. **C.** Violin plots showing the relative expression of the indicated genes in WT and *Ptpn11*^*E76K*/+^ HSCs and GMPs.

Consistent with the regulon results, *Ptpn11*^*E76K*/+^ stem cells and GMPs demonstrated heightened cell cycling, as evidenced by the loss of quiescence (the G_0_ phase in the cell cycle) and an increased number of cells in the G_2_/M phase, based on single-cell transcriptomes and a reported predictor for allocating individual cells to G_0_, G_1_/S, and G_2_/M cell cycle phases^24^ (Fig. 7A). The cell division/replication-related histone H1 family members (*Hist1h1c, Hist1h1d, Hist1h1e*, and *Hist1h2ae*) and CDK1 were upregulated in both *Ptpn11*^*E76K*/+^ stem cells and GMPs (Fig. 7B). Additionally, GSEA revealed a significant enrichment of cell cycling-associated gene sets in *Ptpn11*^*E76K*/+^ stem cells (Fig. 7C). Both *Ptpn11*^*E76K*/+^ stem cells and GMPs exhibited a high enrichment of GM-CSF response gene sets. This observation aligns with the well-established high sensitivity of JMML cells to GM-CSF^25,26^.

### S100a9 and S100a8, aberrantly expressed in Ptpn11^E76K/+^ stem cells, contribute significantly to leukemogenesis.

**Figure 8:**
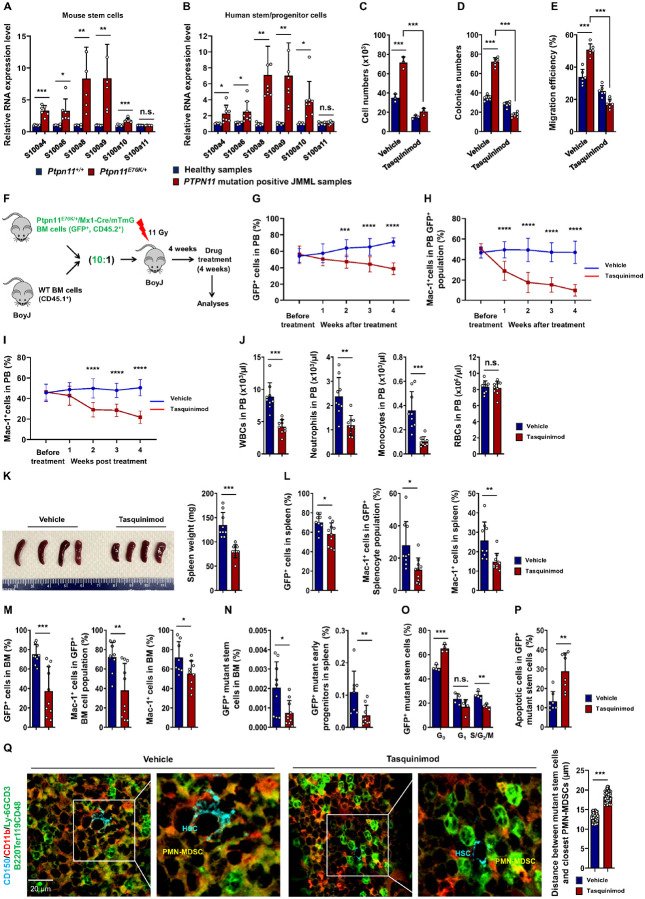
S100a9 and S100a8, aberrantly expressed in *Ptpn11*^E76K/+^ stem cells, contribute significantly to leukemogenesis. **A.** mRNA levels of the indicated genes in the stem cells (Lineage^−^Sca-1^+^c-Kit^+^CD150^+^CD48^−^) sorted from WT and *Ptpn11*^*E76K*/+^ mice were determined by quantitative reverse transcription PCR (qRT-PCR) (n=6 mice/genotype). **B.** CD34^+^ early hematopoietic progenitors isolated from healthy BM samples and *PTPN11* mutated JMML patients were analyzed for mRNA levels of the indicated genes by qRT-PCR (n=5–7 individuals/group). **C.** Stem cells freshly sorted from WT and *Ptpn11*^*E76K*/+^ mice were cultured (5×10^2^ cells) in StemSpan medium supplemented with SCF (50 ng/ml), TPO (50 ng/ml), FLT3-L (50 ng/ml), and tasquinimod (5.0 μM) or vehicle. Seven days later, total cell numbers were determined (n=3 mice/group). **D.** Colonies derived from WT and *Ptpn11*^*E76K*/+^ mutant stem cells (5×10^2^ cells) in the presence of tasquinimod (5.0 μM) or vehicle were determined by CFU-assays as described in Materials and Methods (n=6 mice/group). **E.** Transmigration efficiency of PMN-MDSCs (CD11b^+^Ly6G^+^) toward WT and *Ptpn11*^*E76K*/+^ stem cells in the presence of tasquinimod (5.0 μM) or vehicle were assessed as described in Materials and Methods (n=6 mice/group). **F-Q.** BM cells collected from *Ptpn11*^*E76K*/+^/*Mx1-Cre/mTmG* mice were mixed with WT BM cells from congenic BoyJ mice at a 10:1 ratio and transplanted them into lethally irradiated BoyJ mice (n=13 mice/group). Four weeks post-transplantation, tasquinimod or vehicle was administered to mice via drinking water (5 mg/kg body weight/day) for 4 weeks (**F**). Total leukemic cells (GFP^+^) in the peripheral blood (PB) (**G**), myeloid cells (Mac-1^+^) in the GFP^+^ leukemic cell compartment (**H**) and the entire PB (**I**) were determined at the indicated time points. Mice were euthanized 4 weeks after the treatment. White blood cell counts (WBCs), neutrophils, monocytes, and red blood cell counts (RBCs) in the PB were analyzed (**J**). Spleens were weighted (**K**). Total leukemic cells (GFP^+^), Mac-1^+^ myeloid cells in the GFP^+^ leukemic compartment and the entire spleen and BM were determined (**L**, **M**). The pool size of GFP^+^ stem cells (GFP^+^Lineage^−^Sca-1^+^c-Kit^+^CD150^+^CD48^−^) in the BM, GFP^+^ early progenitors (GFP^+^Lineage^−^Sca-1^+^c-Kit^+^) in the spleen (**N**), cell cycling status (**O**) and apoptosis (**P**) in GFP^+^ stem cells were determined. **Q.** BM sections prepared from tasquinimod- or vehicle-treated *Ptpn11*^*E76K*/+^ mice were immunostained for stem cells (CD150^+^CD3^−^B220^−^Ter119^−^CD48^−^) (cyan) and PMN-MDSCs (CD11b^+^Ly6G^+^) (yellow) (n=10 mice/group, with >76 stem cells/group examined in total). The distance between stem cells and the closest PMN-MDSCs was determined.

Given the prominent upregulation of *S100a9* and *S100a8* in *Ptpn11*^*E76K*/+^ mutant long-term stem cells (Fig. 1G) and their diverse roles in various cell types^27,28^, we investigated their potential role in these tumor initiating cells. First, we confirmed a significant increase (> 8-fold) in the expression levels of S100a9 and S100a8 in mutant stem cells isolated from *Ptpn11*^*E76K*/+^ mice compared to those in WT HSCs (Fig. 8A). Importantly, expression levels of S100a9 and S100a8 were also elevated approximately 7-fold in leukemic stem/progenitor cells (CD34^+^) from *PTPN11*-mutated JMML patients compared to those in normal CD34^+^ hematopoietic stem/progenitors (Fig. 8B). The overexpression of S100a9 and S100a8 by *Ptpn11*^*E76K*/+^ mutant stem cells appeared to promote the growth of these leukemia-initiating cells. *Ptpn11*^*E76K*/+^ stem cells cultured in *ex vivo* expansion medium exhibited significantly accelerated proliferation compared to WT HSCs. However, this growth advantage was mitigated by tasquinimod, an inhibitor of S100a9/S100a8 that disrupts their interactions with receptors RAGE and TLR4^29,30^ (Fig. 8C), which were also highly expressed on these cells (Fig. 1H). Additionally, the elevated differentiation capabilities of *Ptpn11*^*E76K*/+^ mutant stem cells to form myeloid colonies compared to those of WT HSCs were substantially decreased by tasquinimod (Fig. 8D). These findings suggest that S100a9 and S100a8 significantly contribute to the clonal expansion and enhanced myeloid differentiation of leukemia-initiating *Ptpn11*-mutated stem cells through autocrine effects.

Previous studies have proposed a significant role for S100a9 and S100a8 expressed in tumor cells in recruiting MDSCs, which are known for their association with immunosuppression and Inflammation^27,31,32^. These heterogeneous cells co-express CD11b, Ly6G, and Ly6C myeloid lineage markers [polymorphonuclear MDSCs (PMN-MDSCs): CD11b^+^Ly6G^+^Ly6C^low^; mononuclear MDSCs (M-MDSCs): CD11b^+^Ly6G^−^Ly6C^high^]. MDSCs are potent inhibitors of anti-tumor immunity, contributing to immune escape^27,31,32^. To investigate the potential interplay between *Ptpn11*^*E76K*/+^ mutant stem cells and MDSCs, we conducted transwell migration assays. As displayed in Fig. 8E, *Ptpn11*^*E76K*/+^ mutant stem cells demonstrated heightened chemoattracting activities for PMN-MDSCs (CD11b^+^Ly6G^+^) compared to WT HSCs. Notably, this effect was blocked by the S100a9/S100a8 inhibitor tasquinimod, indicating that the overproduction of S100a9 and S100a8 by leukemia-initiating *Ptpn11* mutant stem cells may contribute to the recruitment of MDSCs to the microenvironment.

To test this possibility and further determine the role of S100a9 and S100a8 in the leukemogenic activities of *Ptpn11*^*E76K*/+^ stem cells in an *in vivo* setting, we evaluated the therapeutic impact of the S100a9/S100a8 inhibitor tasquinimod using a widely used transplantation leukemia model. *Ptpn11*^*E76K*/+^/*Mx1-Cre/mTmG* mice were generated by crossbreeding of *Ptpn11*^*E76K/+*^*/Mx1-Cre* mice^6^ with lineage tracing *mTmG* transgenic mice^33^, which expressed red fluorescent protein (RFP) but transitioned to green fluorescent protein (GFP) upon the induction of *Cre* expression (and the *Ptpn11*^*E76K*^ mutation). To mimic clinical scenarios, we combined BM cells from *Ptpn11*^*E76K/+*^*/Mx1-Cre/mTmG* leukemic mice with WT BM cells from congenic BoyJ mice at a 10:1 ratio and transplanted mixed cells into lethally-irradiated BoyJ mice. Four weeks post-transplantation, when donor cells were engrafted, tasquinimod or vehicle was administered to mice via drinking water for 4 weeks (Fig. 8F). Despite the high ratio of leukemic cells in the mixed donor cells, the reconstitution of leukemic cells from *Ptpn11*^*E76K*/+^ mutant stem cells in the recipient mice was approximately 50% due to the hyperactivation and significant depletion of the mutant stem cell population (known as exhaustion) in the BM collected from the leukemic mice^6^. Importantly, in response to tasquinimod treatment, a notable reduction in total leukemic cells (GFP^+^) in the peripheral blood (PB) was observed (Fig. 8G). Myeloid cells (Mac-1^+^) in the GFP^+^ leukemic cell compartment (Fig. 8H) and the entire PB (Fig. 8I) significantly decreased, indicating that the skewed myeloid differentiation of leukemia-initiating *Ptpn11*^*E76K*/+^ stem cells was largely rectified by blocking S100a9/S100a8 function.

Mice were euthanized after 4 weeks of treatment. White blood cell counts (WBCs) in the tasquinimod-treated group significantly decreased, specifically in neutrophils and monocytes, with no apparent changes in red blood cell counts (RBCs) (Fig. 8J). Splenomegaly was also ameliorated in tasquinimod-treated mice (Fig. 8K). Total leukemic cells (GFP^+^) in the spleen, Mac-1^+^ myeloid cells in the GFP^+^ leukemic compartment and the entire spleen all decreased (Fig. 8L). Similar therapeutic effects were also observed in the BM (Fig. 8M). Furthermore, we assessed the impact of the S100a9/S100a8 inhibitor on leukemia-initiating mutant stem cells. As shown in Fig. 8N, GFP^+^ *Ptpn11*^*E76K*/+^ mutant stem cells in the BM and early leukemic progenitor cells (Lineage^−^Sca-1^+^c-Kit^+^) in the spleen significantly decreased in the inhibitor-treated mice. Consistently, the cell cycling of hyperactive *Ptpn11*^*E76K*/+^ stem cells was reduced by the treatment (Fig. 8O). Moreover, apoptosis in these mutant stem cells increased in the inhibitor-treated mice (Fig. 8P), suggesting that S100a9 and S100a8 played an important role for the survival of these leukemia initiating cells. Finally, we visualized *Ptpn11*^*E76K*/+^ stem cells and surrounding cells in tasquinimod- or vehicle-treated mice and found that the distance between these leukemia-initiating cells (CD150^+^CD11b^−^Ly6G^−^CD3^−^B220^−^Ter119^−^CD48^−^) (cyan) and the closest PMN-MDSCs (CD11b^+^Ly6G^+^) (yellow) was significantly increased following tasquinimod treatment (Fig. 8Q), confirming that the recruitment of PMN-MDSCs to the microenvironment of *Ptpn11* mutant stem cells was attributed to S100a9/S100a8 overexpressed by these leukemia-initiating cells.

## DISCUSSION

While considerable progress has been made in understanding the etiology of JMML, numerous questions remain, particularly concerning the cellular and molecular mechanisms that confer a selective advantage to the original leukemia-initiating cells. Understanding these mechanisms can illuminate how leukemia-initiating cells persist in established disease and how these tumor precursor cells may be effectively targeted and eliminated therapeutically. By undertaking a comprehensive characterization of the transcriptomic landscapes across all stages of tumor cell development in *Ptpn11* mutation-associated JMML and substantiating our findings through experimental validation, we have discovered that *Ptpn11*-mutated stem cells (leukemia-initiating cells) are primed by the myeloid transcriptional program and that innate immune and inflammatory responses are aberrantly activated in these cells. These mutant stem cells exhibit strikingly heightened expression of evolutionarily conserved genes that are typically activated in mature myeloid cells during pathogen defense, including anti-microbial peptides (*Camp, Lcn2, Lyz2, Ltf, Chil3*, and *Pglyrp1*) and essential trace metal-sequestering proteins (*S100a9* and *S100a8*), which also function as pro-inflammatory proteins triggering and amplifying innate immune responses^27,28^.

The innate immune system is conventionally activated through the recognition of pathogen-associated molecular patterns (PAMPs) or damage-associated molecular patterns (DAMPs) proteins derived from host cells or damaged cells by pattern-recognition receptors, including TLRs, on myeloid immune cells. These patterns play an important role in recruiting and activating myeloid immune cells, initiating Inflammation to eliminate invading microorganisms^27,28^. S100a9 and S100a8, which show the most significant overexpression in *Ptpn11*^*E76K*/+^ mutant stem cells, are also categorized as DAMPs. They preferentially heterodimerize to form calprotectin, which, like their monomeric/homodimeric forms, are endogenous ligands for TLR4, Rage/Ager, and CD33^21,34,35^ on myeloid effector cells, activating intracellular signaling pathways and culminating in the production of inflammatory cytokines, chemokines, and antimicrobial peptides. Interestingly, the expression of Rage/Ager and CD33 is also markedly elevated on *Ptpn11* mutant stem cells, producing autocrine effects. The autocrine effects of S100a9/S100a8 indeed contributed to the expansion of these leukemia initiating cells (Fig. 8C). Moreover, given the well-characterized detrimental effects of inflammatory challenges on normal HSCs^36,37^, the pro-tumoral inflammatory milieu provides leukemia-initiating mutant stem cells with a competitive advantage over normal counterparts, ultimately resulting in their clonal dominance. S100a9 and S100a8 may also contribute to immune evasion of JMML-initiating mutant stem cells by chemoattracting and expanding immunosuppressive MDSCs in the microenvironment. MDSCs are classically linked to immunosuppression, Inflammation, and cancer, profoundly inhibiting T cell- and NK cell-mediated antitumor immunity through various mechanisms^27,31,32^. S100a9 is crucial for MDSC recruitment as MDSC accumulation in tumors is abolished in S100a9-null mice^38^, and expression of S100a9 in transgenic mice drives expansion and activation of MDSCs^35^. These immune suppressive cells can also secrete abundant S100a9/S100a8 heterodimers, bind to their own surface receptors and nurture an autocrine feedback loop that sustains MDSC recruitment, thereby maintaining immune suppression within the local microenvironment^21^. Moreover, S100a9 also contributes to anti-tumor immunity by inhibiting dendritic cell differentiation^38^. *Ptpn11*^*E76K*/+^ mutant stem cells indeed demonstrate a strong ability to attract MDSCs, and this chemoattracting effect is diminished by the inhibitor of S100a9/S100a8 (Fig. 8E and 8Q). Furthermore, administration of the S100a9/S100a8 inhibitor impedes leukemia development from *Ptpn11*^*E76K*/+^ mutant stem cells (Fig. 8F–8P). These results strongly suggest that the overexpression of S100a9 and S100a8 by *Ptpn11*- mutated stem cells plays a pivotal role in the initial leukemogenic process.

Further investigations are necessary to elucidate how the *Ptpn11*^*E76K*^ mutation instigates a myeloid-specific transcriptional program and co-opts innate immune responses in the mutated stem cells. Shp-2 (encoded by *Ptpn11*) is predominantly localized to the cytosol and plays a prominent positive role in Ras signaling^9,10^. Since other genes that are mutated in JMML are also clustered in the Ras signaling pathway, it is conceivable that the *Ptpn11* mutation causes pathogenic effects mainly through the Ras pathway. However, Shp-2 is also localized to the nucleus and the mitochondrion^39–41^. There is therefore a possibility that the *Ptpn11*^*E76K*^ mutation influences myeloid-specific transcriptomic activities through its nuclear and/or metabolic functions. The role of mutant Shp-2 in different cellular compartments may reveal novel avenues for understanding the diverse molecular mechanisms underpinning the aberrant activation of the myeloid transcriptional program in *Ptpn11*-mutated stem cells. Considering the distinctive subcellular localization of Shp-2 compared to other oncoproteins associated with JMML, it is important to ascertain whether dysregulated innate immune responses are also implicated in other JMML subtypes.

Another noteworthy finding of this study is the dysregulation of ribosomal biogenesis and function in *Ptpn11*^*E76K*/+^ leukemic cells consistently throughout all stages, including leukemia-initiating stem cells. Several ribosomal small and large subunit proteins displayed upregulation in *Ptpn11*^*E76K*/+^ leukemic cells, consistent with the elevated protein translation essential for robust tumor cell growth. Intriguingly, there was a simultaneous decrease in the expression of certain ribosomal proteins. Recent research has revealed the heterogeneity of ribosomes, with different ribosome types displaying preferences for translating specific subsets of mRNAs^22,23^. Diminished expression of ribosomal proteins has the potential to disrupt ribosome formation and function. This can also contribute to malignancies through several mechanisms. The impairment in ribosomes can impact the synthesis of crucial regulatory proteins involved in cell growth, differentiation, and maturation, such as the tumor suppressor p53^42–44^. Moreover, reduced expression of specific ribosomal proteins and perturbed ribosome function can induce chronic ribosomal stress, triggering cellular dysfunctions and genomic instability^22^. However, the precise mechanisms by which the *Ptpn11* mutation selectively interferes with the expression of different ribosomal genes remain unclear.

In summary, our findings reveal previously unappreciated mechanisms in the initial phase of JMML leukemogenesis, where leukemia-initiating mutant stem cells exploit innate immune signaling to gain a selective advantage and evade anti-tumor immunity. The significant dysregulation of proinflammatory proteins S100a9 and S100a8 underscores their pivotal role in orchestrating immune evasion and creating an inflammatory microenvironment conducive to leukemic progression. This insight offers new perspectives for developing therapeutic strategies to disrupt leukemia-initiating stem cells and improve treatment outcomes in JMML.

## MATERIALS AND METHODS

### Mice.

*Ptpn11*^*E76K* Neo/+^ conditional knock-in mice were generated in our previous study^6^. *Mx1-Cre*^*+*^ (Strain #: 003556) ^45^, mTmG dual-fluorescent reporter transgenic mice (Strain #: 007676)^33^, C57BL/6 mice (CD45.2^+^) (Strain #: 000664), and BoyJ mice (CD45.1^+^) (Strain #: 002014) were purchased from the Jackson Laboratory. All mice were kept under specific-pathogen-free conditions at Emory University Division of Animal Resources. Animal procedures complied with the NIH Guidelines for the Care and Use of Laboratory Animals and were approved by the Institutional Animal Care and Use Committee.

### Patient specimens.

De-identified samples from *PTPN11*-mutated patients with JMML and pediatric healthy controls normal BM biopsies were obtained from the University of California, San Francisco and the Aflac Cancer and Blood Disorders Center Biorepository of Children’s Healthcare of Atlanta. Samples were obtained after written, informed consent under locally approved institutional review board research protocols and in accordance with the Declaration of Helsinki.

### Single-cell transcriptome profiling.

Fresh BM cells were collected and pooled from *Ptpn11*^*E76K/+*^*/Mx1-Cre*^*+*^ mice and *Ptpn11*^*+/+*^*/Mx1-Cre*^*+*^ control mice (3 mice/group), followed by the execution of the recommended protocol for the scRNA-seq 10x Genomics platform using v3 chemistry. In brief, scRNA-seq raw reads were obtained following the standard protocol for Chromium Single Cell 3 Reagent Kits v3. Subsequently, the CellRanger 1 software from 10x Genomics was employed to identify cell-discriminating barcode sequence markers and unique molecular identifier (UMI) markers for different mRNA molecules within each cell. This process aimed to quantify the high-throughput single-cell transcriptome and conduct data quality statistics and comparisons against the original genome. Next, the Seurat 2 software package was utilized for further quality control (QC) and processing of the CellRanger results. In the QC step, delocalized cells were filtered by fitting a generalized linear model. Subsequently, the distribution of nUMI (unique molecular identifier counts), nGene (number of detected genes), and percent.mito (percentage of mitochondrial genes) was assessed to filter out low-quality cells, such as double cells, multiple cells, or dead cells, leaving only qualified cells for further bioinformatics analyses.

### t-distributed stochastic neighbor embedding (t-SNE) visualization and cell identification.

The single-cell transcriptome underwent principal component analysis (PCA) for linear dimensionality reduction. Subsequently, the PCA results were visualized in a two-dimensional space using t-SNE, a non-linear dimensionality reduction technique. The Seurat platform’s FindAllMarkers function was employed to identify marker genes for each cell classification relative to other cell populations. These identified genes serve as potential markers for each cell type. Visualization of the identified marker genes was carried out using the VlnPlot and FeaturePlot functions. Following the clustering process, the Single R platform was utilized to assign cell types based on published datasets^19,20^, thereby enhancing the accuracy of cell type classification.

### Gene set enrichment analysis (GSEA).

GSEA was conducted to identify genes associated with specified cell types such as HSCs (Hematopoietic Stem Cells) and GMPs (Granulocyte-Macrophage Progenitors). The analysis utilized the GSEA platform available at http://www.broadinstitute.org/gsea/index.jsp. To prepare input data for GSEA, the top 5000 variable genes in each group were selected using the Seurat “FindVariableGenes” function. Gene sets, including those from KEGG pathways and Gene Ontology (GO), were obtained from the molecular signatures database (MSigDB).

### Single-cell regulatory network inference and clustering (SCENIC) analysis.

SCENIC analyses were performed using version 1.1.2.2, corresponding to RcisTarget 1.2.1 and AUCell 1.4.1. The motifs database for RcisTarget and GRNboost was utilized with default parameters. In detail, the analysis involved identifying over-represented transcription factor binding motifs on a given gene list using the RcisTarget package. Subsequently, the AUCell package was employed to score the activity of each group of regulons in each cell. This process enabled the inference and clustering of regulatory networks at the single-cell level, offering insights into the regulatory landscape of the analyzed cell populations.

To evaluate the cell type specificity of each predicted regulon, the regulon specificity score (RSS) was computed, employing the Jensen-Shannon divergence (JSD) as a measure of similarity between two probability distributions. Specifically, the JSD was calculated for each vector of binary regulon activity overlaps with the assignment of cells to specific cell types. The connection specificity index (CSI) for all regulons was determined using the scFunctions package, accessible at https://github.com/FloWuenne/scFunctions/.

### Pseudotime analysis.

We utilized the Monocle2 package (v2.9.0) for inferring cell differentiation trajectories. The specific steps were as follows: First, we employed the importCDS function from the Monocle2 package to convert the Seurat object to the CellDataSet object. Next, the differentialGeneTest function was utilized to filter out ordering genes (genes with a q-value < 0.01). Then, we used the reduceDimension function to perform dimensionality reduction clustering. Finally, we applied the orderCells function to infer the differentiation trajectory.

### Cell-cell communication analysis.

We utilized CellPhoneDB (v2.0) to identify biologically relevant ligand-receptor interactions from single-cell transcriptomic data. We defined a ligand or receptor as ‘expressed’ in a particular cell type if 10% of the cells of that type exhibited non-zero read counts for the ligand/receptor encoding gene. Statistical significance was assessed by randomly shuffling the cluster labels of all cells and repeating the aforementioned steps, thereby generating a null distribution for each ligand-receptor (LR) pair in each pairwise comparison between two cell types. Following 1,000 permutations, *p*-values were calculated using the normal distribution curve generated from the permuted LR pair interaction scores. To delineate networks of cell-cell communication, we connected any two cell types where the ligand was expressed in the former cell type and the receptor in the latter. The R package circlize was employed for visualizing the cell-cell communication networks.

### Fluorescence-activated cell sorting (FACS) analysis and cell sorting.

FACS analyses were performed on a Cytoflex flow cytometer (Beckman Coulter Life Sciences), following standard procedures. For HSC staining, BM cells were harvested, washed, and incubated for 30 min at 4°C in phosphate buffered saline (PBS) with 2% fetal bovine serum (FBS) containing the following antibodies: anti-Mac-1 PerCP/Cyanine5.5 (Biolegend, 101228, clone M1/70),anti-Gr-1 Pacific Blue (Biolegend,108430, clone RB6–8C5), anti-Ter119 PE (Biolegend, 116208, clone TER-119), anti-B220 PE (eBiosciences, 12-0452-83, clone RA3–6B2), anti-CD3 PE (BD Biosiences Pharmingen, 553064, clone 145–2C11), anti-Mac-1 PE (Biolegend, 101208, clone M1/70), anti-Gr-1 PE (eBiosciences, 12-5931-83, clone RB6–8C5), anti-Scal-1 PE/Cyanine7 (Biolegend, 108114, clone D7), anti-c-Kit APC/Cyanine7 (Biolegend, 105826, clone 2B8), anti-CD48 Percp (eBioscience, 46-0481-80, clone HM48–1), anti-CD150 AF647 (Biolegend, 115918, clone TC15–12F12.2). HSCs were defined as Lin^−^Sca-1^+^c-Kit^+^CD150^+^CD48^−^. For apoptosis analyses, fresh BM cells were stained for HSCs, and then incubated with Annexin V-BV605 (BD Biosiences Pharmingen, 563974, clone Annexin V) (0.7 μg/ml) and 4’,6-diamidino-2-phenylindole (DAPI) (0.3 μg/mL). For the cell cycle analysis, fresh BM cells were stained for HSCs as above, fixed and permeabilized using a Cytofix/Cytoperm kit (BD Biosciences). The samples were then stained with Ki-67 BV605 (Biolegend, 652413) and further incubated with Hoechest 33342 (20 μg/ml). Data were collected on a Beckman Coulter CytoFLEX flow cytometer and analyzed with FlowJo (Tree Star). For cell sorting, BM cells were first lineage-depleted using a lineage depletion kit. Cells were then stained with fluorochrome-labeled antibodies. Sorting of specific cell populations was conducted using BD FACSAia II following standard gating strategies.

### Colony-forming unit (CFU) assay.

Freshly sorted HSCs (5×10^2^ cells) were plated in triplicate in 35-mm dishes in 0.9% methylcellulose IMDM medium containing 15% FBS, Gln (10^− 4^ M), β-mercaptoethanol (3.3×10^− 5^ M), SCF (50 ng/ml), IL-3 (20 ng/ml), IL-6 (20 ng/ml), and EPO (3 Units/ml). After 12 days of incubation at 37°C in 5% CO2, myeloid colonies derived were counted under an inverted microscope.

### Transmigration assay.

Transmigration assays were conducted with 5.0 μm pore transwells (Corning). Briefly, HSCs freshly sorted from WT and *Ptpn11*^*E76K*/+^ mice were suspended in in StemSpan media (STEMCELL Technologies) containing 20% FBS, 50 ng/mL SCF, 50 ng/mL Flt3L, 20 ng/mL IL-3, and 20 ng/mL IL-6. Six hundred microliters of cell suspension (2×10^3^ cells) were loaded to lower chambers. The S100a9/S100a8 inhibitor tasquinimod was then added to the chamber (5.0 μM). CD11b^+^Ly6G^+^ myeloid cells freshly sorted from normal C57BL6 mice were labeled with carboxyfluorescein succinimidyl ester (CFSE) (1.0 μM), washed and resuspended at 1×10^6^ cells/ml in the same medium as that in lower chambers but without the inhibitor. One hundred microliters of cell suspension were added to upper chambers. Cells were allowed to migrate across the membrane at 37°C in 5% CO_2_ for 2 hours. Both input cells, cells collected from the upper chamber, and cells collected from the lower chambers were analyzed by FACS. Migration efficiency was then calculated.

### Immunofluorescence staining.

Tissue sections were prepared from paraffin-embedded mouse femurs, deparaffinized, and rehydrated following standard protocols. The slides were stained with the following antibodies following standard procedures: anti-CD150 AF647 (Biolegend, 115918, clone TC15–12F12.2), anti-CD11b PE (Biolegend, 101208, clone M1/70), anti-Ly-6G AF488 (Biolegend, 127625, clone 1A8), anti-Ter119 FITC (Biolegend, 116206, clone TER-119), anti-CD3 FITC (Biolegend, 100306, clone 145–2C11), anti-B220 FITC (Biolegend, 103206, clone RA3–6B2), and anti-CD48 FITC (Biolegend, 103403, clone HM48–1) antibodies. Images were acquired using Leica Stellaris 8 and processed with ImageJ 1.54f software.

### Statistics and reproducibility.

Unless otherwise noted, data are presented as mean ± SD of biological replicates (independent animals/independent experiments) (n numbers are shown on graphics or specified in figure legends). Unpaired two-tailed Student’s t-test was used for the statistical comparison of the two groups. * *p* < 0.05; ** *p* < 0.01; *** *p* < 0.001, **** *p* < 0.0001.

## Data Availability

The raw scRNA-seq data generated in this study have been deposited in the Gene Expression Omnibus database under accession code GSE266821.
